# Timeliness and accuracy of the 7-Item Japan Urgent Stroke Triage (JUST-7) score, a prehospital stroke triage tool, assessed by emergency medical services

**DOI:** 10.1371/journal.pone.0309326

**Published:** 2024-08-22

**Authors:** Takayuki Nishiwaki, Yukiko Enomoto, Yusuke Egashira, Hirofumi Matsubara, Takamitsu Hori, Nozomi Sasaki, Takahiro Yoshida, Noriyuki Nakayama, Naoyuki Ohe, Shinji Ogura

**Affiliations:** 1 Department of Neurosurgery, Gifu University Graduate School of Medicine, Gifu, Japan; 2 Department of Emergency and Disaster Medicine, Gifu University Graduate School of Medicine, Gifu, Japan; Foshan Sanshui District People’s Hospital, CHINA

## Abstract

The prompt initiation of stroke treatment significantly influences patient outcomes, highlighting the crucial role of prehospital triage. This study aimed to assess the implementation of the 7-Item Japan Urgent Stroke Triage (JUST-7) score by emergency medical services (EMS) in our region and its effect on emergency transportation for suspected stroke patients. Data were collected from patients suspected of having an acute stroke with a Cincinnati Prehospital Stroke Scale (CPSS) score of 1 or more who were transferred by ambulance within 24 h of symptom onset. Two prehospital stroke scales were employed during different periods: period 1 with CPSS alone (January to December 2020) and period 2 with both CPSS and JUST-7 (January 2021 to March 2023). On-scene time data were obtained from the EMS crews, and data regarding the final diagnosis of patients and their outcomes were obtained from the respective hospitals to which the patients were transferred. These data were compared between periods 1 and 2 and between the CPSS and JUST-7. The results revealed that additional evaluation with JUST-7 did not affect ambulance transport time. The CPSS+JUST-7 approach demonstrated higher specificity in identifying stroke and major artery occlusion than with the CPSS alone; however, an appropriate cut-off value needs to be considered. The JUST-7 achieved a diagnostic concordance rate of 35.9% for the most likely stroke type and 64.0% for the first two most likely types. This research emphasizes the potential of JUST-7 as a valuable addition to prehospital stroke diagnosis protocols. Its flexibility in adapting cut-off values based on regional factors and available medical resources optimizes its utility in diverse healthcare settings. The JUST-7 score is a promising tool for improving patient outcomes through prompt and accurate prehospital assessments.

## Introduction

Mechanical thrombectomy for large-vessel occlusion (LVO) is increasingly recommended based on substantial evidence from landmark clinical trials, such as MR CLEAN [1], ESCAPE [2], EXTEND-IA [3], SWIFT PRIME [4], and REVASCAT [5]. Prompt treatment initiation and minimization of the time from symptom onset to recanalization have a significant impact on patient outcomes [6]. Consequently, rapid prehospital triage to an appropriate hospital and rapid initiation of treatment are of utmost importance.

In Japan, the social stroke network system, including the certification of primary stroke centers (PSCs) and the establishment of a comprehensive stroke acceptance system, has been facilitated following the recommendations of the American Stroke Association (ASA). However, a prehospital triage system for stroke patients has not been established in Japan, and emergency medical services (EMS) in our region use only the Cincinnati Prehospital Stroke Scale (CPSS) [7]. To ensure appropriate prehospital triage by EMS, the development of a reliable pre-hospital scale for accurately predicting stroke is crucial.

The 7-Item Japan Urgent Stroke Triage (JUST-7) [8] score incorporates seven specific items to calculate the probability of each stroke type. In the present study, we evaluated the implementation of the JUST-7 score using EMS in our region and its effect on emergency transportation in patients with suspected stroke. We further investigated the diagnostic performance of JUST-7 for stroke and LVO. This study is unique in its prospective examination of the diagnostic ability of the JUST-7 in a real-world setting and is novel in that it compares the JUST-7 to the CPSS.

## Materials and methods

### Study population

Patients transported by ambulance with suspected acute stroke within 24 h of the onset of symptoms with a CPSS of 1 or higher in the Gifu City Medical Area were included in the present study. We divided the study period into two per the prehospital stroke scale used by the EMS crew (period 1, CPSS alone from January 2020 to December 2020; and period 2, a combination of CPSS with JUST-7 from January 2021 to March 2023). During this period, stricter measures to prevent the spread of COVID-19 were declared twice in our medical area (April 2020 to May 2020, and April 2021 to September 2021).

### Gifu City Medical Area

The Gifu City Medical Area comprises Gifu City and its surrounding cities and falls under the jurisdiction of the Gifu City Fire Department. It covers an area of 833.6 km^2^ and serves a target population of 535,292. The aging rate in this region is approximately 28%, which is close to the national average for Japan. Within this medical area, patients were transported to 13 hospitals, comprising two PSC core facilities equipped for 24-h thrombectomy procedures, seven PSC hospitals with 24-h recombinant tissue plasminogen activator (rtPA) capabilities, and four hospitals without a stroke center designation where physicians capable of diagnosing stroke were available 24 h a day. The locational relationship between Gifu City Medical Area and each hospital is shown in S1 Fig.

### JUST-7 score

The JUST-7 is a prehospital scale that uses seven evaluation items for stroke patient triage, providing a rapid assessment of stroke types. It was developed by Uchida et al. [8], and evaluates the probability of major vessel occlusion, intracerebral hemorrhage, subarachnoid hemorrhage (SAH), other infarctions, and non-stroke conditions. The seven items included in the JUST-7 score include a systolic blood pressure of 165 mmHg or more, arrhythmia, gaze deviation, headache, speech disturbance, consciousness disturbance, and upper limb weakness. A multivariable logistic regression model, consisting of these seven items, assigns a specific score to each item based on its individual contribution to the overall prediction of stroke types and then provides a breakdown of the probability percentage for stroke and each stroke type, totaling 100%. The details are described in S2 Fig. The EMS crews used mobile devices to evaluate seven items and calculate the JUST-7 score. All EMS crews involved in this study belonged to the Gifu City Fire Department and were already skilled in CPSS evaluation. Prior to the initiation of this study, they underwent training on using the JUST-7 assessment method. Following the prehospital scale results, the EMS crews discussed suitable hospital candidates and made transportation requests.

### Data collection

We accessed the collected data in April 2023 and assessed the following information from the EMS crews: on-scene time (the duration from EMS contact with patients to transfer to the hospital), transportation time (the duration from deciding on the transport hospital to arrival at the hospital), patient’s age and sex, and the positive items on each scale (CPSS and JUST-7) evaluated by the EMS crew. The EMS crew inputted the positive items into each scale programmed on a mobile device at the scene. The output from the JUST-7, which included the stroke probability and the probability of each stroke type, was recorded in the electronic data captured (EDC) by the EMS crews. In each case, the most probable stroke type in the JUST-7 scale was defined and recorded as the prehospital diagnosis, and the second probable stroke type was defined and recorded as the prehospital differential diagnosis. The representative cases for these definitions are shown in S2 Fig.

The final diagnosis was made by neurologists or neurosurgeons at the transfer centers using MRI or CT scans. Subsequently, the final diagnoses of the four-stroke types in the JUST-7 mentioned above and diagnoses other than stroke were recorded in the electronic medical records and transferred to EDC for analysis. EDC data for all cases are presented in supplemental data (S1 Dataset).

### Outcomes

The outcomes were the effects of newly adding JUST-7 to CPSS on emergency transport times as well as whether the addition of JUST-7 can enhance the diagnostic abilities of stroke and LVO. The diagnostic abilities were evaluated by sensitivity, specificity, and Youden index, as well as by comparing the prehospital diagnoses in JUST-7 with the final diagnoses.

### Statistical analysis

For a comparative analysis between the two groups (the CPSS alone group and the CPSS + JUST-7 group), appropriate statistical tests were employed based on the nature of the data and distribution. Independent t- test was used for continuous variables such as age. Chi- squared or Fisher’s exact tests were used for categorical variables, such as male sex and positive items, on the scale to examine the between-group associations. Linear regression analysis was performed on on-scene time and transportation time using age, male sex, number of items subject to the CPSS, positive items on the CPSS (limb paralysis, facial paralysis, and speech disturbance), and final diagnosis as covariates.

The sensitivity (the ratio of true positives to the sum of true positives and false negatives) and specificity (the ratio of true negatives to the sum of true negatives and false positives) for stroke and LVO were measured. In terms of CPSS, cases that met a certain number of items or more were diagnosed prehospitally as stroke or LVO, and sensitivity and specificity were calculated. For JUST-7, all cases with a particular calculated probability or higher were diagnosed prehospitally as stroke or LVO, regardless of the disease type in the prehospital diagnosis, and sensitivity and specificity were calculated. The Youden index (summing the sensitivity and specificity) was added up to find the ideal cut-off value for each scale. Regarding only LVO, we also calculated the sensitivity and specificity when diagnosing LVO in the first or second place in the ranking among possible stroke types in the JUST-7.

Additionally, a correct diagnosis was defined as a case in which the final diagnosis matched the prehospital diagnosis, and the rate was calculated for all cases and for each stroke type. A correct differential diagnosis was defined as a case where the final diagnosis was included in either the prehospital diagnosis or differential diagnosis, and this rate was also calculated.

All statistical analyses were conducted using JMP version 10 (SAS Institute, Cary, NC), and p-value less than 0.05 were considered statistically significant.

All study procedures were approved by the local Institutional Review Board of our institute, Medical Review Board of Gifu University Graduate School of Medicine and informed in writing in advance and verbal consent was obtained in verbal for the use of detailed patient data.

## Results

The study included 109 eligible patients in the CPSS-alone group (period 1) and 256 in the CPSS + JUST-7 group (period 2) (Table 1). There were no significant differences in age (CPSS alone: 74.3 ±12.2 years vs. CPSS + JUST-7: 75.1±13.7 years, p = 0.60) and male ratios (CPSS alone: 59.6% vs. CPSS + JUST-7: 53.1%, p = 0.25) between the two groups. Table 1 shows the number of applicable CPSS items, the proportion of neurological findings, and the final diagnosis in each group. Despite the absence of significant differences in the number of CPSS items and neurological findings between groups, the CPSS + JUST-7 group demonstrated a higher rate of LVO than the CPSS-alone group (43.4% vs. 30.3%, p = 0.019) at the final diagnosis. The rate of other infarctions was also significantly lower in the CPSS + JUST-7 group (7.03% vs. 16.5%, p<0.05).

**Table 1:** Patient characteristics (age, male sex, patient neurological findings, and final diagnosis) of each group.

	All patients	CPSS alone	CPSS + JUST -7	P-value^a^^,^ ^b^^,^ ^c^
**No. of patients**	365	109	256	N/A
**Age (years)**	74.8 ± 13.2	74.3 ± 12.2	75.1 ± 13.7	0.60^a^
**Male sex (n (%))**	201 (55.1)	65 (59.6)	136 (53.1)	0.25^b^
**No. of items subject to CPSS**				
0 (n (%))	30 (8.22)	13 (11.9)	17 (6.64)	0.092^c^
1 (n (%))	88 (24.1)	23 (21.1)	65 (25.4)	0.381^c^
2 (n (%))	129 (35.3)	34 (31.2)	95 (37.1)	0.279^c^
3 (n (%))	118 (32.3)	39 (35.8)	79 (30.9)	0.358^c^
**CPSS items**				
Limb paralysis (n (%))	280 (76.7)	86 (78.9)	194 (75.6)	0.589^b^
Facial paralysis (n (%))	152 (41.6)	44 (40.4)	108 (42.2)	0.747^b^
Speech disturbance (n (%))	268 (73.4)	78 (71.6)	190 (74.2)	0.599^b^
**No. of items subject to JUST-7**				
0 (n (%))	N/A	N/A	1 (0.39)	N/A
1 (n (%))	N/A	N/A	15 (5.86)	N/A
2 (n (%))	N/A	N/A	52 (20.3)	N/A
3 (n (%))	N/A	N/A	78 (30.5)	N/A
4 (n (%))	N/A	N/A	67 (26.2)	N/A
5 (n (%))	N/A	N/A	30 (11.7)	N/A
6 (n (%))	N/A	N/A	12 (4.69)	N/A
7 (n (%))	N/A	N/A	1 (0.39)	N/A
**JUST-7 items**				
sBP ≥ 165mmHg (n (%))	N/A	N/A	165 (64.5)	N/A
Arrhythmia (n (%))	N/A	N/A	67 (26.2)	N/A
Gaze deviation (n (%))	N/A	N/A	61 (23.8)	N/A
Headache (n (%))	N/A	N/A	37 (14.5)	N/A
Speech disturbance (n (%))	N/A	N/A	175 (68.4)	N/A
Consciousness disturbance (n (%))	N/A	N/A	149 (58.2)	N/A
Upper limb weakness (n (%))	N/A	N/A	196 (76.6)	N/A
**Final diagnosis**				
Subarachnoid hemorrhage (n (%))	15 (4.11)	6 (5.51)	9 (3.52)	0.395^a^
Large vessel occlusion (n (%))	144 (39.5)	33 (30.3)	111 (43.4)	0.019^a^
Other infarction (n (%))	36 (9.86)	18 (16.5)	18 (7.03)	0.007^a^
Cerebral hemorrhage (n (%))	150 (41.1)	47 (43.1)	103 (40.2)	0.608^a^
Other stroke (n (%))	20 (5.48)	5 (4.59)	15 (5.86)	0.803^a^

CPSS, Cincinnati Prehospital Stroke Scale; JUST– 7, 7-Item Japan Urgent Stroke Triage; sBP, systolic blood pressure; N/A, not applicable

^a^ P-values were calculated using Student’s t-test

^b^ P-values were calculated using Chi- squared test

^c^ P-values were calculated using Fisher’s exact test

The average time spent on-scene by the EMS crews was 11.91 ± 3.8 minutes in the CPSS alone group and 11.77 ± 3.3 minutes in the CPSS + JUST-7 group. Additionally, the average transportation time was 12.61 ± 7.0 minutes in the CPSS alone group and 11.74 ± 6.5 minutes in the CPSS + JUST-7 group. Linear regression analysis revealed that adding the JUST-7 score by the EMS crews had no significant impact either on the on-scene time (predicted values: -0.13 minutes, p = 0.52) or the transportation time (predicted values: -0.41 minutes, p = 0.30) (Table 2).

**Table 2:** Impact of adding the 7-Item Japan Urgent Stroke Triage (JUST-7) and other factors on pre-hospital spending time.

	On-scene time (min)				Transportation time (min)			
Covariates	Predicted values	SEM	95% CI	p value	Predicted values	SEM	95% CI	p value
**Adding JUST-7**	-0.13	0.20	-0.53–0.27	0.52	-0.41	0.40	-1.18–0.37	0.30
**Age**	0.02	0.01	-0.01–0.05	0.16	0.03	0.03	-0.02–0.09	0.24
**Male sex**	0.31	0.19	-0.07–0.69	0.11	-0.16	0.38	-0.91–0.58	0.67
**Limb paralysis**	0.28	0.26	-0.23–0.79	0.28	0.30	0.51	-0.69–1.29	0.55
**Facial paralysis**	0.15	0.35	-0.54–0.84	0.67	0.47	0.69	-0.89–1.82	0.50
**Speech disturbance**	0.31	0.25	-0.19–0.81	0.23	-0.40	0.50	-1.38–0.58	0.42
**No. of items subject to CPSS**								
**1**	-0.15	0.27	-0.68–0.37	0.57	0.23	0.52	-0.80–1.26	0.66
**3**	-0.64	0.39	-1.40–0.13	0.10	-0.08	0.77	-1.58–1.43	0.92
**Final diagnosis**								
**Subarachnoid hemorrhage**	0.56	0.84	-1.08–2.21	0.50	1.14	1.64	-2.08–4.37	0.49
**Large vessel occlusion**	-0.24	0.38	-0.98–0.50	0.53	-1.45	0.74	-2.91–<-0.01	0.05
**Other infarction**	-1.31	0.54	-2.37–-0.24	0.02	-1.02	1.06	-3.10–1.06	0.34
**Cerebral hemorrhage**	-0.07	0.37	-0.80–0.67	0.86	0.27	0.74	-1.18–1.71	0.71

CPSS, Cincinnati Prehospital Stroke Scale; JUST– 7, 7-Item Japan Urgent Stroke Triage; SEM, Standard Error of the Mean; 95%CI, 95% Confidence Interval

The sensitivity and specificity for stroke varied with the number of CPSS items as follows: 1 item, 92.2% and 15.79%; 2 items, 69.95% and 68.42%; and 3 items, 33.53% and 89.47%. Using a cut-off value of 46.3% for the stroke probability of the JUST-7, the sensitivity was almost comparable to that of the CPSS 1 item (94.2% vs. 92.2%). However, the JUST-7 demonstrated higher specificity (35.71% vs. 15.79) (Table 3).

**Table 3:** Comparison between the Cincinnati Prehospital Stroke Scale (CPSS) and 7-Item Japan Urgent Stroke Triage (JUST-7) in stroke diagnostic ability.

No of items subject to CPSS	Sensitivity (%)	Specificity (%)	Youden index
**1 item or more**	92.2	15.79	107.99
**2 items or more**	69.65	68.42	138.07
**3 items**	33.53	89.47	123
**Probability of stroke in JUST-7 scale**			
**30.10%**	99.2	15.24	114.4
**40.20%**	95	28.57	123.6
**46.30%**	94.2	35.71	129.9
**55.90%**	88	42.1	130.1
**88.30%**	47.1	71.43	118.5
**96.30%**	13.2	92.86	106.1

CPSS, Cincinnati Prehospital Stroke Scale; JUST– 7, 7-Item Japan Urgent Stroke Triage

The rate of correct diagnosis was 35.9%, which is synonymous with considering the most likely stroke type in the JUST-7 as the final diagnosis. Additionally, the correct differential diagnostic rate was 64.0%, which is synonymous with considering the first two most likely stroke types (Table 4). The correct diagnosis rate by stroke type was observed to be high for infarctions other than major artery occlusion (88.9%), whereas it was comparatively low for SAH (11.1%).

**Table 4:** The rate of correct diagnosis and differential diagnosis between the prehospital diagnosis and differential diagnosis made with the 7-Item Japan Urgent Stroke Triage (JUST-7) and the final diagnosis.

Final diagnosis of subtypes of stroke	Correct diagnosis rate	Correct differential diagnosis rate
**Subarachnoid hemorrhage**	1/9 (11.1%)	2/9 (22.2%)
**Large vessel occlusion**	25/111 (22.5%)	61/111 (55.0%)
**Other infarction**	16/18 (88.9%)	17/18 (94.4%)
**Cerebral hemorrhage**	45/103 (43.7%)	78/103 (75.7%)
**Other stroke**	5/15 (33.3%)	6/15 (40.0%)
**Total**	92/256 (35.9%)	164/256 (64.0%)

For major artery occlusion, the specificity of the CPSS remained low despite the increasing number of applicable items: 1 item, 10.86%; 2 items, 34.84%; and 3 items, 68.33%. Conversely, with a cut-off value of 19.16% probability on the JUST-7 score, the specificity improved and surpassed that of the CPSS with 3 items (70.34% vs. 68.33%) while maintaining a reasonably high sensitivity of 54.95%. The Youden index (125.29) was the highest in this scenario. Alternatively, considering major artery occlusion as positive if it ranked second in the JUST-7 probable stroke types, the Youden index was high (124.61), and the diagnostic ability was similar to the cut-off value of 19.16% (Table 5).

**Table 5:** Comparison between the diagnostic ability of the Cincinnati Prehospital Stroke Scale (CPSS) and 7-Item Japan Urgent Stroke Triage (JUST-7) in identifying large-vessel occlusion.

No. of items subject to CPSS	Sensitivity(%)	Specificity (%)	Youden index	Positive predictive value	Negative predictive value
**1 item or more**	95.83	10.86	106.96	41.19	80
**2 items or more**	71.53	34.84	106.37	41.7	65.25
**3 items**	33.33	68.33	101.66	40.68	61.13
**Probability of major artery occlusion in JUST-7**					
**10%**	66.67	49.66	116.33	50.34	66.06
**13.95%**	61.26	57.93	119.19	52.71	66.14
**17.31%**	57.66	62.07	119.73	53.78	65.69
**19.16%**	54.95	70.34	125.29	58.65	67.11
**20%**	49.55	71.72	121.27	57.29	65
**30%**	23.42	88.28	111.7	60.47	60.09
**50%**	10.81	94.48	105.29	60	58.05
**Ranking of major artery occlusion among probable stroke types in the JUST-7**					
**First most probable**	17.36	92.31	109.67	59.52	63.16
**By the second probable**	54.95	69.66	124.61	58.1	66.89

CPSS, Cincinnati Prehospital Stroke Scale; JUST-7, 7-Item Japan Urgent Stroke Triage

## Discussion

This study was conducted to evaluate the impact of adding the JUST-7 to the CPSS for prehospital stroke diagnosis on ambulance transport and the stroke diagnostic accuracy of JUST-7, primarily focusing on major artery occlusion. The addition of the JUST-7 had no effect on ambulance transport time and demonstrated superior specificity for stroke and major artery occlusion compared to the CPSS. To the best of our knowledge, this is the first report to assess the impact of integrating the JUST-7 within the realm of ambulance transportation and to validate stroke prediction rates and stroke types in real-world settings.

Acute cerebral infarction is a disease in which treatment delay is not an option because time significantly affects the patient’s outcome. A 30-min delay in starting intravenous t-PA therapy and cerebrovascular therapy (treatments for acute ischemic stroke) can result in a 10% reduction in the patient’s favorable outcome rate [6]. Given the well-established efficacy of acute thrombectomy, the spectrum of eligible cases is expanding, and recanalization rates are improving owing to technological advancements and the maturation of surgical techniques [9, 10]. While the number of patients who derive treatment benefits is steadily increasing, individuals transported to facilities unequipped for endovascular treatment might necessitate subsequent transportation, potentially resulting in missed treatment opportunities or a lack of symptom improvement, even after receiving treatment. Kollikowski et al. reported that the prehospital transport pathway influences the progression of cerebral infarction [11]. Hence, in acute major artery occlusion, decisions made during emergency transportation have a substantial influence on patient outcomes.

The CPSS is a widely used prehospital stroke assessment tool [7]. Furthermore, other tools, such as ELVO [12], GAI2AA2 [13], and the JUST-7 [8] have demonstrated efficacy for pre- or in-hospital triage of LVO. In contrast to the CPSS, these scales are more intricate because of their greater number of evaluation items, some of which involve non-visual assessments, such as unilateral spatial neglect. Araki et al. reported the effect of introducing the JUST score, which evaluates 21 items, in Hiroshima City and found no impact on on-scene or transportation times. They also noted an increase in the acceptance rate of the initial request and a decrease in door-to-puncture times. However, no improvement in patient outcomes was observed [14]. In our study, the introduction of the JUST-7, which features a more carefully selected set of evaluation items than the JUST score and a reduced number of evaluation components, did not impact on-scene activity or transport times. This can be attributed to the streamlined data input process facilitated by mobile devices and the inclusion of several evaluation items, reducing the time spent at the scene. Moreover, the clear categorization of each predicted disease type simplifies the decision-making process for selecting an appropriate hospital for patient transportation.

The JUST-7 is the only stroke scale capable of predicting stroke, including its subtypes. In the present study, the predictive value for each disease type was highest for other cerebral infarctions and lowest for SAH. Other cerebral infarctions, such as lacunar infarctions, mainly involve mild symptoms and are clearly distinguishable from cerebral hemorrhages and major artery occlusions that exhibit distinct cortical symptoms. Regarding SAH, the number of cases was relatively limited in this study population, as it targeted patients who were positive for at least one item of the CPSS during an emergency call, primarily focusing on cases with focal neurological symptoms. Consequently, the number of cases was limited, potentially contributing to the lower hit rate of SAH. Because the probability of each type is expressed as a probability (up to 100%), relying solely on the diagnosis with the highest value may lead to the inadvertent exclusion of slightly lower-value types. The hit rate was approximately 60% when diseases with the second-highest probability were considered. Hayashi et al. used machine learning to create a prehospital diagnostic algorithm for stroke subtypes and reported a superior predictive value compared to the JUST-7 [15]. Although achieving more accurate predictions may be possible by incorporating deep learning in the future, the JUST-7 appears to be the most straightforward method for EMS.

The JUST-7 possesses the distinctive attribute of providing the probability of each disease as a like continuous value. Consequently, when utilizing it for prehospital screening, determine the optimal cut-off value is imperative. When dealing with a stroke, prioritizing sensitivity is paramount. Therefore, the CPSS 1 item is commonly used for the initial screening of suspected stroke cases. With respect to the JUST-7, when the stroke probability threshold was set at 46.2%, a sensitivity equivalent to that of CPSS 1 item was achieved, and the JUST-7 exhibited superior specificity. Further reducing the cut-off probability has been suggested for possibly attaining a sensitivity greater than that of the CPSS. In the context of LVO, the peak sum of sensitivity and specificity (AUC, 0.62) was 19.16%. Smith et al. reported in a meta-analysis that none of the 19 LVO prediction scales had high sensitivity or specificity [16]. This also holds true for the JUST-7. However, its strength lies in its ability to continuously adjust the sensitivity and specificity by changing the threshold, enabling customization to regional needs. For instance, in regions with limited medical resources, there may be a preference for prioritizing specificity, resulting in a higher cut-off value for prediction, whereas in areas with ample medical resources, there may be a preference for prioritizing sensitivity, leading to a lower cut-off value. The JUST-7 is regarded as unique in its flexibility in accommodating these variations.

In a comparison in the literature with ELVO and GAI2AA [12, 13], which are frequently used in Japan, the specificity for LVO was similar between the JUST-7 and ELVO (JUST-7: 70.3%, ELVO: 72%), but it was not as high as for GAI2AA (81%). Furthermore, the sensitivity was highest in the order of GAI2AA, ELVO, and JUST-7 (91% vs 85% vs 55%). This is because the JUST-7 includes non-cortical symptoms such as convulsions, blood pressure, and headaches, whereas ELVO and GAI2AA are scales created mainly based on cortical symptoms. This is thought to be because the scale was created for only LVO screening. If the screening focuses on LVO, it may be better to use ELVO (pre-hospital) + GAI2AA (in-hospital). Nevertheless, if diagnosing stroke subtypes is important in a given region, JUST-7 may have an advantage.

The present study had several limitations. First, patient selection was based on the information provided during the emergency call, primarily relying on the patient’s chief complaint. In some cases, the emergency call may be conducted by someone other than the patient, which could lead to inaccuracies in symptom description. Second, the initial assessments were conducted by EMS personnel. Although they have received comprehensive training, variations may still be present in their evaluation abilities. Third, this study was conducted within a limited area of the Gifu City Medical Area. Regional differences in demographics and emergency transportation systems may have affected the generalizability of the findings.

Despite these limitations, this study is the first to implement the JUST-7 in a real-world setting. Future research should delve into regional variations in cut-off values to tailor prehospital stroke diagnosis to specific healthcare landscapes.

## Conclusion

The integration of the JUST-7 into a real-world setting did not prolong patient transport time. Notably, the JUST-7 outperformed the CPSS in stroke type prediction, suggesting its potential as a valuable addition to prehospital stroke diagnosis protocols. Furthermore, considering the second-highest disease prediction alongside primary diagnosis seems to enhance its utility. However, recognizing that the optimal cut-off value for LVO prediction may vary depending on regional factors and available medical resources is important for further assessing the adaptability of the JUST-7.

## Supporting information

S1 Fig Geography of Gifu prefecture, Gifu City Medical Area, and local relationships with the hospitals involved in this study. (TIF)

S2 Fig The evaluation items of CPSS and JUST-7, and the commonalities and differences between each scale and an example of JUST-7 results. (TIF)

S1 Dataset All data obtained from patients and emergency medical services in this study. (XLSX)
