# Supplementary figures and images for: Timeliness and accuracy of the 7-Item Japan Urgent Stroke Triage (JUST-7) score, a prehospital stroke triage tool, assessed by emergency medical services
Source: PLoS One. 2024 Aug 22;19(8):e0309326. doi: 10.1371/journal.pone.0309326 (PMC11340938; doi:10.1371/journal.pone.0309326)

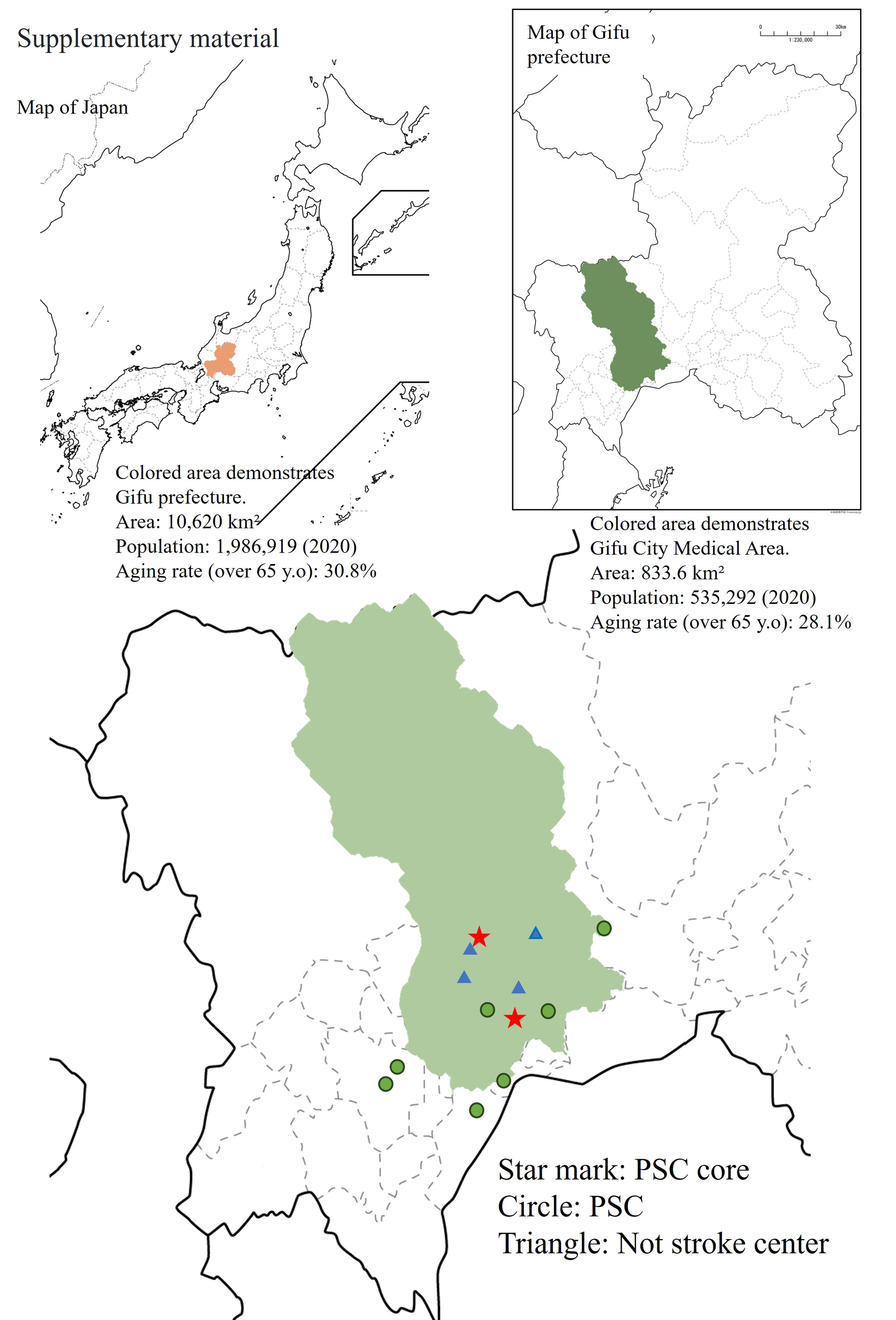

Supplement: S1 Fig — (TIF) [file pone.0309326.s001.tif]

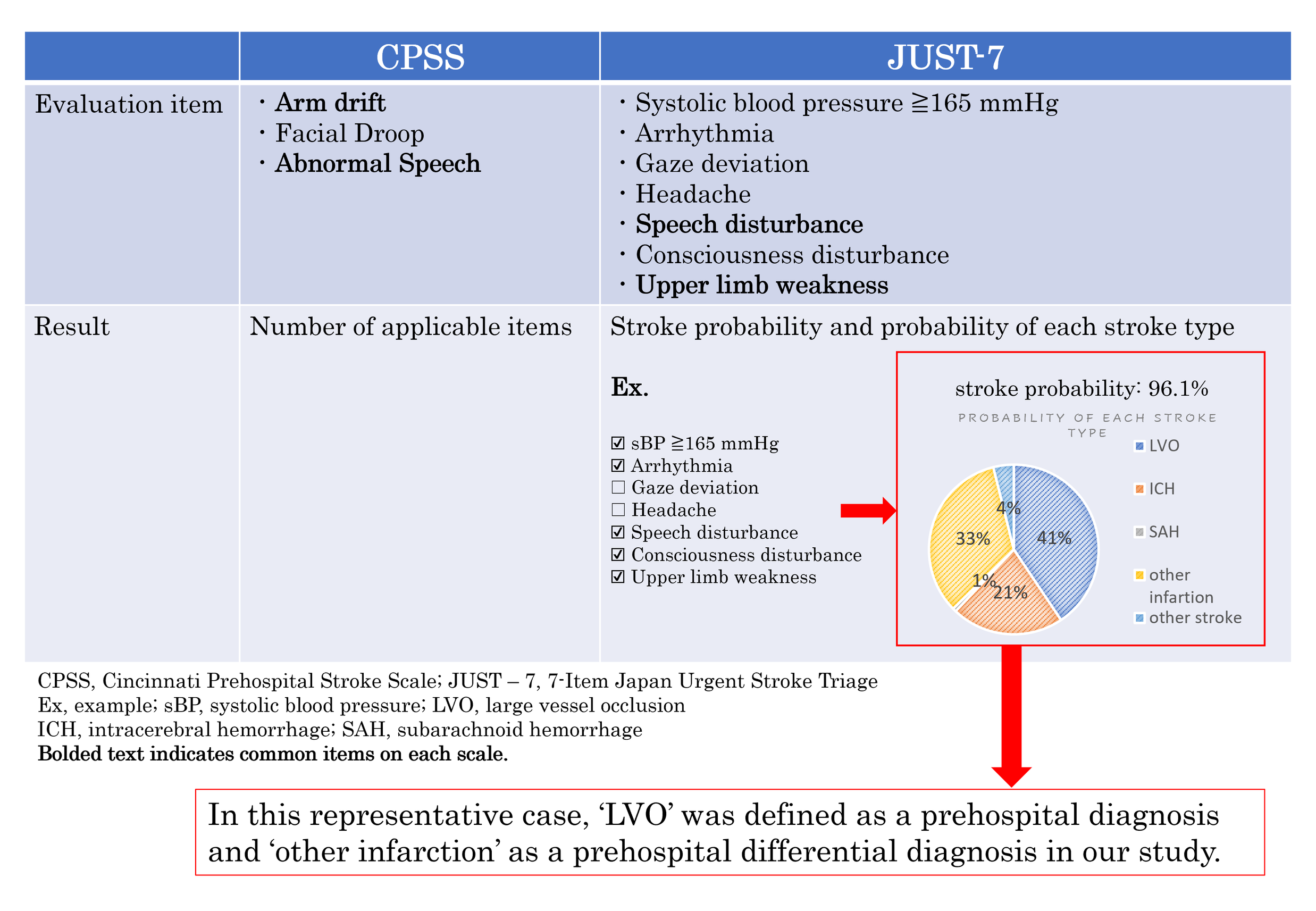

Supplement: S2 Fig — (TIF) [file pone.0309326.s002.tif]
